# Formation and Characterisation of Posaconazole Hydrate Form

**DOI:** 10.3390/ph16010065

**Published:** 2022-12-31

**Authors:** Michail Lykouras, Malvina Orkoula, Christos Kontoyannis

**Affiliations:** 1 Department of Pharmacy, University of Patras, GR-26504 Rio, Achaias, Greece; 2 Institute of Chemical Engineering Sciences, Foundation of Research and Technology-Hellas (ICE-HT/FORTH), GR-26504 Platani, Achaia, Greece

**Keywords:** posaconazole, polymorphism, Form-S, hydrate, oral suspension, X-ray powder diffraction (XRPD), Raman spectroscopy, attenuated total reflection (ATR), optical microscopy, thermal analysis

## Abstract

Posaconazole is an API added as Form I for the production of oral suspensions, but it is found as Form-S in the final formulation. In this study, it was found that this polymorphic conversion, which may affect the bioavailability, is due to an interaction with water. However, the relatively poor wettability of posaconazole Form I renders the complete wetting of its particles and production of pure Form-S challenging. Consequently, for its isolation, Form I should be dispersed in water followed by application of sonication for at least 10 min. Pure posaconazole Form-S was characterised using X-ray powder diffraction (XRPD), Raman spectroscopy, attenuated total reflection (ATR) spectroscopy, thermogravimetric analysis (TGA) and optical microscopy. From these techniques, posaconazole Form-S was characterised as a hydrate form, which includes three molecules of water per API molecule.

## 1. Introduction

Pharmaceutical liquid oral suspensions consist of two phases—an internal phase, containing the dispersed active pharmaceutical ingredient (API) and an external phase, known also as the continuous phase, which is referred to as the liquid dispersion medium. Water is selected as the external phase in the majority of the pharmaceutical oral suspensions. The major drawback of these pharmaceutical formulations is the lack of physical stability [1]. The production of physically stable oral suspensions requires the proper wetting of the API particles and the careful control of their sedimentation rate. If the hydrophobicity of the API is too high for the particles to be wet, they tend to aggregate, forming porous clumps, which are dispersed in the dispersion medium or float on the surface of the suspension. In order to avoid pure wettability, surfactants are added in the oral suspensions, which decreases the interfacial tension among the solid particles and the dispersion medium molecules, lowering the contact angle [1,2,3,4]. Good wettability of the dispersed API particles also prominent results in a low sedimentation rate. According to Stoke’s law, the velocity of sedimentation of the dispersed particles is proportional to the particle size and the difference between the densities of the dispersed phase and the continuous phase, while it is inversely proportional to the viscosity of the dispersion medium [1,5]. Therefore, good wettability leads to an acceptable sedimentation rate of the solid particles, as the possibility of particle size growth due to the formation of aggregates or lowering the density of the dispersed phase because of porous clumps is reduced. The rheological properties of the suspension are affected by pure wetting of the API particles, as well [6]. The size of the particles and their wettability may also be affected by a change in the polymorphism of the API, resulting in a lack of stability of the oral suspension [7].

Polymorphism, the ability of a specific chemical compound or substance to exist in more than one crystal form, retaining its chemical properties [8,9,10], could be responsible for significant alterations in the efficacy, effectiveness and safety of the APIs [11]. Therefore, the control of API polymorphism in their pharmaceutical formulations during manufacture, storage and shelf-life is crucial.

Posaconazole, among the latest members of triazole antifungal drugs [12], is commercially available under the brand name Noxafil^®^ as a 40 mg/mL oral suspension, as a 18 mg/mL sterile solution for intravenous administration (300 mg concentrate for solution for infusion) and as 100 mg delayed-release gastro-resistant tablets [13,14]. It is administered to treat a number of fungal diseases either as a first-line treatment or as an alternative treatment when the most common antifungal medicines, such as amphotericin B and itraconazole, are not suitable or have failed. Aspergillosis, coccidioidomycosis, mycetoma, fusariosis and oropharyngeal candidiasis are listed among the indications of posaconazole. This broad-spectrum antifungal agent is also licensed for the prophylaxis of invasive aspergillosis and candidiasis in severely immunosuppressed patients who are receiving treatments for hematopoietic stem cell transplantation or hematologic cancers with neutropenia after chemotherapy [12,13,15,16,17]. Its action is expressed through the inhibition of 14α-demethylase, an essential enzyme for the synthesis of ergosterol, which is crucial for the formation of the cell membrane of the fungi [12,13,16,17,18,19].

Until recently, fourteen different forms of posaconazole have been mentioned in the literature. Particularly, the crystalline posaconazole Forms I [20], II [20], III [20], IV [21], V [22], II-S [23], Y [24], A [25], S [26] and N [26] and posaconazole solvates with isopropanol [26], methanol [27] and a mixture of dioxane and water [27] have been found in various patents and studies. The amorphous form of posaconazole has been stated in a couple of patents [22,25,26,28] as well. Among these polymorphic forms and solvates of posaconazole, crystalline Form I is mainly used as raw material for manufacturing posaconazole oral suspensions [29]. However, it has already been demonstrated that a conversion of posaconazole Form I to Form-S is observed in the oral suspensions [30]. The reasons for this polymorphic transition, though, have not been elucidated previously, while no other information for posaconazole Form-S is available except for its XRPD pattern [26,30].

The aim of this study is to shed light on the causes of the polymorphic conversion of posaconazole API in the oral suspensions and investigate the role of water in this transition. A further objective of this study is to find out the conditions under which posaconazole Form I is transformed to posaconazole Form-S. This study also focuses on the preparation and the isolation of pure posaconazole Form-S and its characterisation using techniques that are capable of identifying different crystal forms [31,32,33,34,35]. The techniques used in an effort to elucidate the crystal form of the Form-S are X-ray powder diffraction (XRPD), Raman spectroscopy, and attenuated total reflection (ATR) spectroscopy [31,33], as well as thermal analytical techniques, such as thermogravimetric analysis (TGA) [32]. Optical microscopy was also employed to record images of the crystal form [34].

## 2. Results

### 2.1. Investigation of Posaconazole Form I Transformation

**Figure 1:**
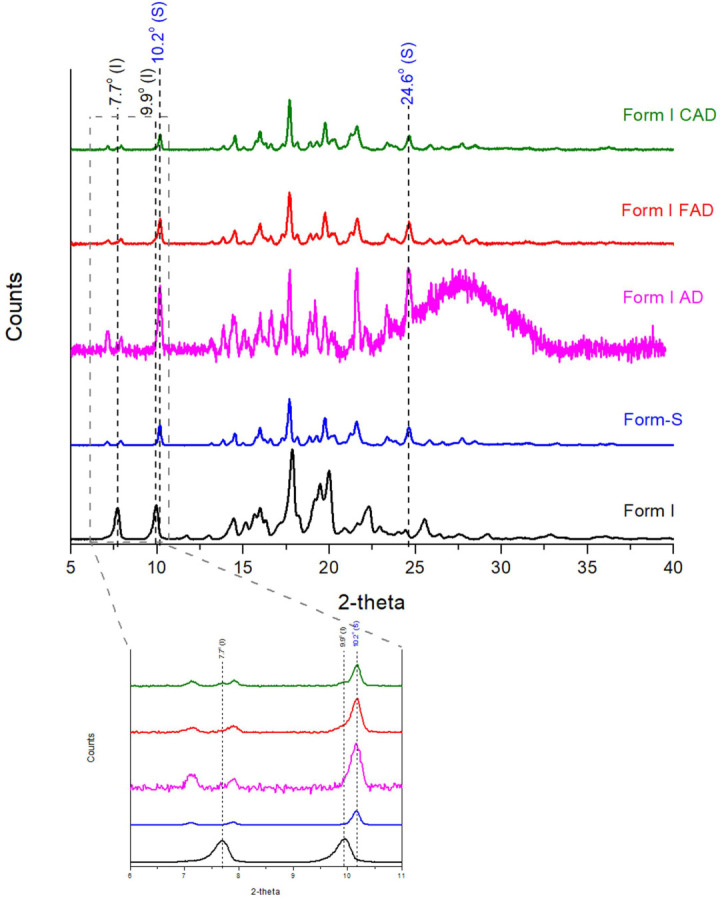
XRPD patterns of Poscaonazole API Form I, posaconazole API Form-S, a posaconazole Form I aqueous dispersion (AD), a posaconazole Form I filtered aqueous dispersion (FAD) and a posaconazole Form I centrifuged aqueous dispersion (CAD) covered with a transparent LDPE cling film (black peak labels: characteristic peaks for posaconazole Form I; blue peak labels: characteristic peaks for posaconazole Form-S).

Posaconazole Form I, which is the thermodynamically stable polymorph of posaconazole, is mainly used for the production of posaconazole oral suspensions. Nevertheless, in the oral suspensions, posaconazole is detected as Form-S, as already mentioned in the introductory section, which was converted back to Form I upon evaporation of the liquid constitiuents [30]. In order to investigate the reasons for this polymorphic transition in the oral suspensions, posaconazole API Form I was mixed with each excipient, separately, in the exact ratios used for the production of posaconazole oral suspensions (Table 1) [14]. The mixtures were prepared by stirring at 500 rpm for 30 min and loaded on appropriate XRPD sample holders. The samples were covered with transparent low-density polyethylene (LDPE) cling film before their XRPD patterns were recorded, so that water evaporation from the sample would be prevented. Posaconazole Form I was stable in all mixtures apart from the mixture of posaconazole Form I with water. The characteristic peaks of posaconazole Form-S at 10.2° and 24.6° 2-theta, as described in our previous study [30] were detected (Figure 1 Form I AD). Therefore, the only excipient responsible for the polymorphic transition of posaconazole Form I to posaconazole Form-S was found to be water. However, the transformation was not complete and Form I was found to co-exist with Form-S (Figure 1-Form I AD).

In order to obtain a better S/N ratio for studying the transformation, a 40 mg/mL aqueous dispersion of posaconazole Form I was prepared by magnetic stirring at 500 rpm for 30 min. Subsequently, the water dispersion was either centrifuged at 8000 rpm, 25 °C for 23 min or filtered using vacuum. In this way, the majority of the excess water molecules were removed and the API was isolated. The XRPD patterns of the posaconazole API received by centrifugation or filtration of posaconazole Form I aqueous dispersion were recorded after being covered with LDPE cling film I order to avoid water evaporation and conversion back to Form I (Figure 1 Form I FAD and Form I CAD). The XRPD patterns were found to highly resemble the pattern of the aqueous dispersion (Figure 1 Form I AD). The isolated posaconazole API was detected as mainly Form-S, since the characteristic peaks of Form-S at 10.2° and 24.6° 2-theta were present in the XRPD patterns. posaconazole Form I was also detected as indicated by its characteristic peaks at 7.6° and 9.8° 2-theta. The process was repeated in triplicate and presence of Form I was found in all XRPD patterns but the intensity of Form I peaks varied considerably, i.e., the transformation percentage was not stable.

Since the percentage of the transformation varied from trial to trial, the influence of the wetting process of posaconazole Form I in the preparation of pure Form-S was studied. The wettability of posaconazole Form I was low as implied by the high value of the contact angle. The contact angle of posaconazole Form I was measured using the sessile drop method [36] and it was determined equal to 75.3° ± 3.8°. Thus, the relatively poor wettability of posaconazole Form I affected the wetting of all API particles and consequently, the complete transformation to Form-S.

### 2.2. Investigation of the Mixing Method for the Production of Pure Posaconazole Form-S

In order to achieve complete conversion to Form-S, the appropriate method of mixing Form I with type II water should be determined, so that all the particles would be wetted. For this purpose, aqueous dispersions of posaconazole Form I were prepared by manual shaking for 1 min or 5 min, vortexing for 1 min, 2 min or 5 min, magnetic stirring at 250 rpm, 500 rpm, 750 rpm or 1000 rpm for 30 min, magnetic stirring at 1000 rpm for 5 min, 15 min, 30 min or 60 min or sonication for 1 min, 2 min, 5 min, 10 min or 15 min. After each sample preparation method, the respective aqueous dispersion was filtered under vacuum through 0.22 μm pore size nitrocellulose filters. The precipitates were loaded on a XRPD sample holder, covered with LDPE cling film and, subsequently, their XRPD patterns were recorded.

Manual shaking led to immediate wetting of the greatest part of API particles, even though some particles agglomerated before their wetting. Similarly, vortex of the aqueous dispersion resulted in a small fraction of agglomerates, which hindered the complete conversion to Form-S. Magnetic stirring using low rotation rates or short times of stirring resulted in agglomeration of a great number of API particles and thus, the conversion to Form-S was not possible. However, even when a 1000 rpm stirring rate was applied for more than 30 min, there was a lack of repeatability in the complete conversion to pure Form-S because, in some cases, Form I particles adhered to the glass vial hindering their wetting. It was only when sonication was applied on the aqueous dispersions for at least 10 min that it was possible to produce pure Form-S (Table 2). This was because sonication was able to break the agglomerates, allowing the complete wetting of posaconazole Form I particles. Eventually, sonication for 15 min was selected for the production of the 40 mg/mL posaconazole Form I aqueous dispersion and the subsequent isolation of pure posaconazole Form-S through its filtration.

### 2.3. Characterisation of Posaconazole Form-S

#### 2.3.1. X-ray Powder Diffraction (XRPD)

The XRPD patterns of posaconazole Form-S and Form I were recorded using a slow scan rate of 4 s/step and compared against each other (Figure 1 Form I and Form-S). A slight shift of ±0.1° 2-theta was observed in the XRPD characteristic peaks of Form I and Form-S regarding to the 2-theta values observed in our previous study. More specifically, the characteristic peaks of posaconazole Form I were found +0.1° 2-theta shifted, i.e., at 7.7° and 9.9° 2-theta, while the characteristic peaks of posaconazole Form-S were detected at 10.2° and 24.6° 2-theta (Figure 1 Form I and Form-S).

##### Determination of the Crystallographic Data of Posaconazole Form-S through XRPD

The peaks of the XRPD patterns of posaconazole Form I and Form-S were identified and they were indexed to Miller indices (hkl) using the dichotomy method [37] as applied by the software PreDICT (International Centre for Diffraction Data, ICDD, Newton Square, PA, USA) [38,39]. No solution was found in the cubic, tetragonal, orthorhombic or hexagonal crystal systems neither for posaconazole Form I nor for posaconazole Form-S. However, for both posaconazole polymorphs, a solution with acceptable M_20_ (de Wolff) [40] and F_20_ (Smith-Snyder) [41] Figure of Merits (FoM) was determined for the monoclinic crystal system (Table 3).

**Figure 2:**
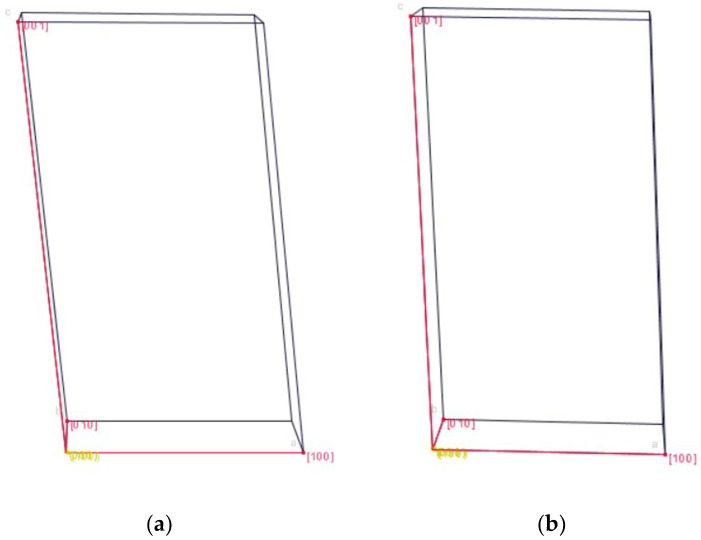
Unit cells of (**a**) posaconazole Form I; (**b**) posaconazole Form-S, as determined via the dichotomy method applied on their XRPD patterns and designed using the JCrystal software.

In the Form-S reduced unit cell, edge a is slightly smaller, while edge c is slightly larger than the respective dimensions of Form I. Edge b of both posaconazole polymorphs is very similar to each other. However, angle β of the Form-S unit cell is smaller than the respective angle of the Form I unit cell (Table 3). Thus, the monoclinic unit cell of posaconazole Form-S has a somewhat orthorhombic shape (Figure 2).

##### Stability of Posaconazole Form-S through XRPD

**Figure 3:**
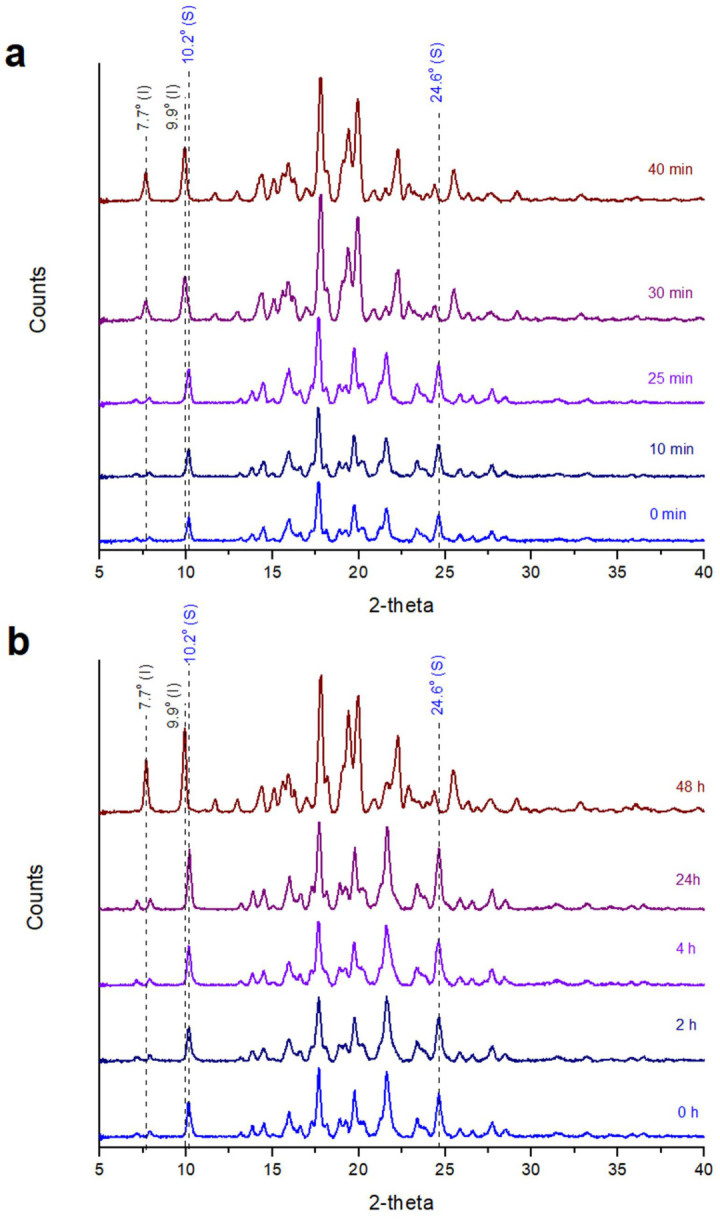
XRPD patterns of posaconazole Form-S left in ambient conditions (**a**) without using a cover; (**b**) using LDPE cling film as a cover (black peak labels: characteristic peaks for posaconazole Form I; blue peak labels: characteristic peaks for posaconazole Form-S).

When posaconazole Form-S was left in ambient conditions, its characteristic peaks at 10.2° and 24.6° 2-theta were detected for 25 min after its isolation via filtration, but they were not observed after 30 min. At the same time, the characteristic peaks of posaconazole Form I at 7.7° and 9.9° 2-theta were not observed during the first 25 min but they fully emerged at the 30 min interval (Figure 3a).

On the contrary, when LDPE cling film was employed in order to delay water evaporation and the subsequent conversion to Form I, posaconazole Form-S peaks were detectable even 24 h since the isolation of pure Form-S, while the transformation to Form I was completed 48 h after the filtration (Figure 3b).

#### 2.3.2. Raman Spectroscopy

**Figure 4:**
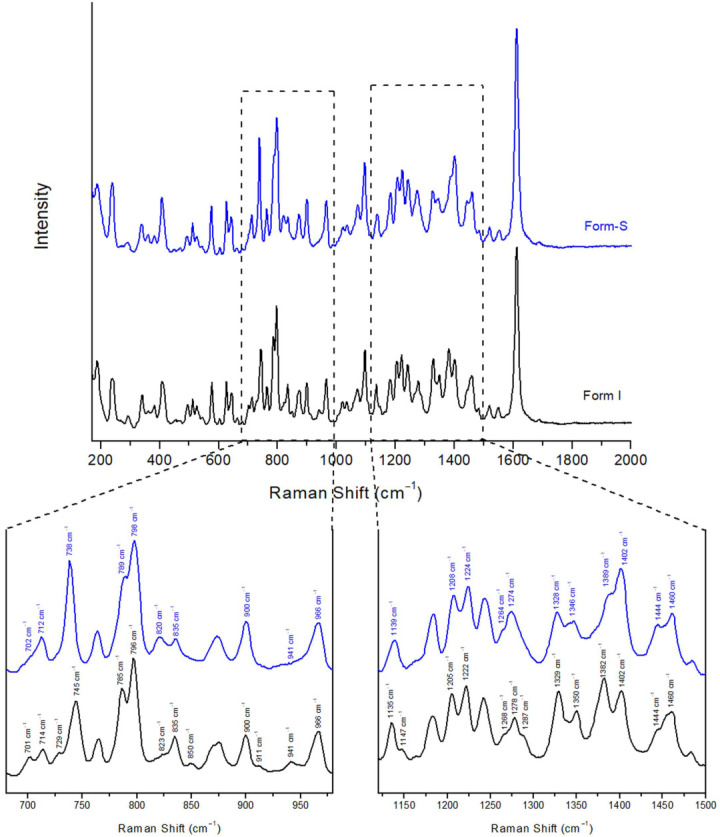
Raman spectra of posaconazole Form I and posaconazole Form-S. Inset: magnification of spectral areas 680–980 cm^−1^ and 1120–1500 cm^−1^ (black peak labels: characteristic peaks for posaconazole Form I; blue peak labels: characteristic peaks for posaconazole Form-S).

For the acquisition of the Raman spectrum of Form-S, the precipitate from the filtration was spread on a gold-coated glass slide and the slide was covered with a layer of LDPE cling film. The Raman spectrum of Form-S was compared against the spectrum of Form I (Figure 4). The most prominent differences between the two spectra are summarised in Table 4.

Multiple slight shifts in peaks, as well as a couple of significant differences, were observed between the two posaconazole polymorphs. The most pronounced differences were observed in the spectral range of 729–745 cm^−1^ and 1382–1402 cm^−1^ (Figure 4). The peak of Form-S at 738 cm^−1^ was well differentiated from the peak of Form I at 745 cm^−1^, which could be used for the characterisation of posaconazole polymorph as Form-S. In addition, the combination of the shoulder at 1389 cm^−1^ and the peak at 1402 cm^−1^ observed in the Raman spectrum of Form-S instead of a double peak at 1382 cm^−1^ and 1402 cm^−1^ detected in the Raman spectrum of Form I could be used for verifying the presence of Form-S and the absence of Form I (Table 4).

##### Stability of Posaconazole Form-S through Raman Spectroscopy

**Figure 5:**
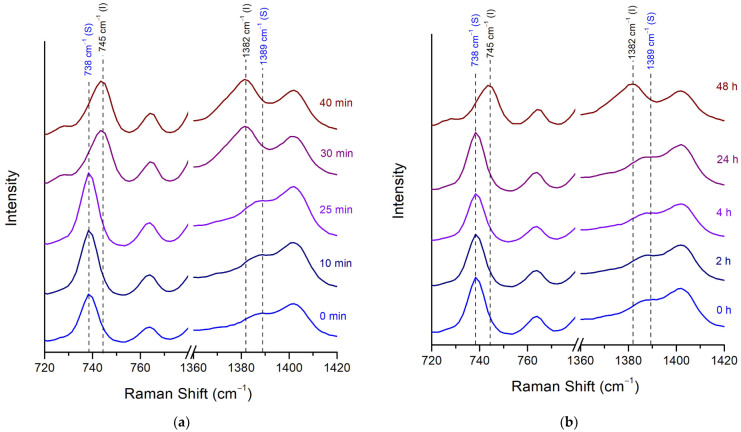
Raman spectra of posaconazole Form-S left in ambient conditions (**a**) without using a cover; (**b**) using LDPE cling film as a cover (black peak labels: characteristic peaks for posaconazole Form I; blue peak labels: characteristic peaks for posaconazole Form-S). Spectral areas: 720–780 cm^−1^ and 1360–1420 cm^−1^.

The stability of pure Form-S in ambient conditions was also studied through Raman spectroscopy with and without the use of LDPE cling film. It was found that the characteristic peak and shoulder of Form-S at 738 cm^−1^ and 1389 cm^−1^, respectively, were present in the Raman spectrum for less than 30 min after the isolation of pure Form-S, when no membrane was applied on the API. After 30 min only the characteristic peaks of Form I at 745 cm^−1^ and 1382 cm^−1^ were detected (Figure 5a). However, the stability of Form-S was significantly increased to more than 24 h when a LDPE cling film was employed (Figure 5b).

#### 2.3.3. Attenuated Total Reflection (ATR) Spectroscopy

**Figure 6:**
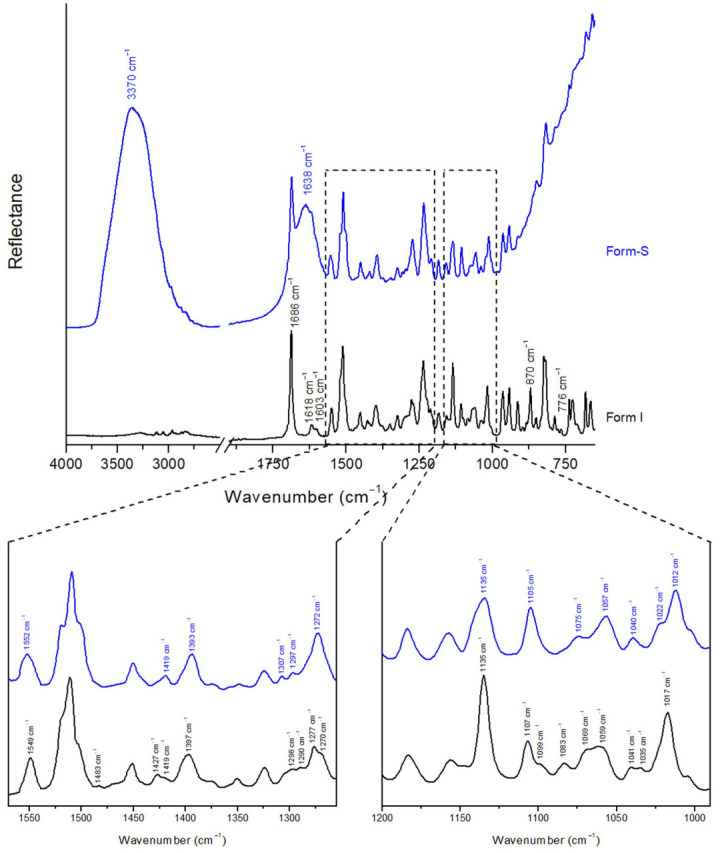
ATR spectra of posaconazole Form I and posaconazole Form-S. Inset: magnification of spectral areas 1570–1255 cm^−1^ and 1200–990 cm^−1^ (black peak labels: characteristic peaks for posaconazole Form I; blue peak labels: characteristic peaks for posaconazole Form-S).

The posaconazole Form-S ATR spectrum was also recorded and is presented along with the ATR spectrum of posaconazole Form I (Figure 6). Form-S, the API was placed on the ATR crystal immediately after its isolation via filtration and it was covered with LDPE film. The acquired ATR spectrum was a combination of the ATR spectra of Form-S and water. The existence of water was evident because of the broad peaks at 3800–2700 cm^−1^ attributed to the stretching vibrations of the -OH group, at 1770–1570 cm^−1^ due to the bending vibrations of water molecules and at 900–650 cm^−1^ due to the stretching and bending vibrations of hydrogen bonds, which are developed among water molecules (Figure 6) [42,43]. The peaks of water in the ATR spectrum of posaconazole Form-S are due to the remaining water molecules in the API after filtration.

Various minor peak shifts and some more prominent differences were detected in the ATR spectrum of Form-S compared to that of Form I (Figure 6). The peak at 1012 cm^−1^ with the shoulder at 1022 cm^−1^ is characteristic for Form-S, while Form I is characterised by a peak at 1017 cm^−1^. Moreover, the presence of the double peak at 1075 cm^−1^ and 1057 cm^−1^ is representative for posaconazole Form-S, whereas in the ATR spectrum of Form I, a broad peak from 1069 cm^−1^ to 1059 cm^−1^ was observed (Table 5).

##### Stability of Posaconazole Form-S through ATR Spectroscopy

**Figure 7:**
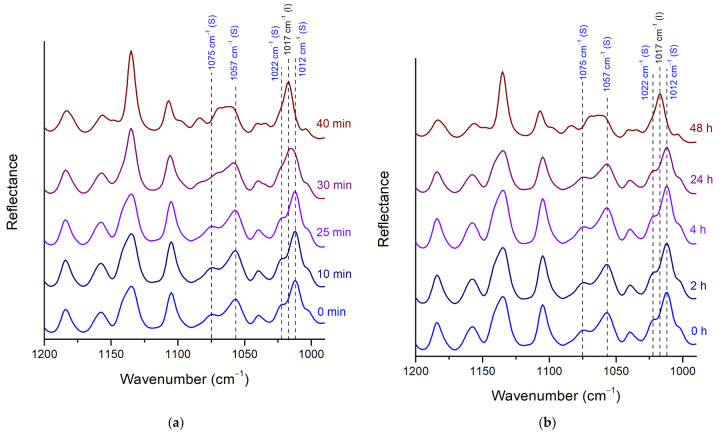
Stability of pure posaconazole Form-S in ambient conditions investigated through ATR spectroscopy (**a**) without using a cover; (**b**) using LDPE cling film as a cover (black peak labels: characteristic peaks for posaconazole Form I; blue peak labels: characteristic peaks for posaconazole Form-S). Spectral area: 1200–990 cm^−1^.

Except for the XRPD and the Raman spectroscopy, the stability of pure posaconazole Form-S in ambient conditions with and without the application of the LDPE membrane was studied via ATR. When no membrane was used, the characteristic peaks of Form-S at 1075 cm^−1^, 1057 cm^−1^ and 1012 cm^−1^ and the shoulder at 1022 cm^−1^ were observed 10 min and 25 min after its isolation, while the peak of Form I at 1017 cm^−1^ and the broad peak at 1069–1059 cm^−1^ were absent. However, after 30 min, the peak of Form-S at 1012 cm^−1^ was shifted to 1015 cm^−1^, while the peaks of Form-S at 1012 cm^−1^ and the shoulder at 1022 cm^−1^ appeared as shoulders of the peak at 1015 cm^−1^. At the same time the shape of the double peak of Form-S at 1075 cm^−1^ and 1057 cm^−1^ changed to a broader peak between 1069 cm^−1^ and 1059 cm^−1^, similar to the broad peak of Form I. Thus, 30 min after the isolation of Form-S, partial polymorphic transition to Form I was observed. The conversion of Form-S back to Form I was completed 40 min after the isolation of Form-S, as it was confirmed by the presence of the peak at 1017 cm^−1^ and the absence of the characteristic peaks of Form-S (Figure 7a).

When the LDPE cling film was employed for the protection of the polymorphic conversion, posaconazole Form-S was stable for more than 24 h, as implied by the double peak at 1075 cm^−1^ and 1057 cm^−1^ and the peak at 1012 cm^−1^ with the shoulder at 1022 cm^−1^. However, in the ATR spectrum recorded 48 h after the isolation of Form-S, only the characteristic peak of Form I at 1017 cm^−1^ was observed, as well as the broad peak at 1069–1059 cm^−1^ (Figure 7b).

#### 2.3.4. Optical Microscopy

**Figure 8:**
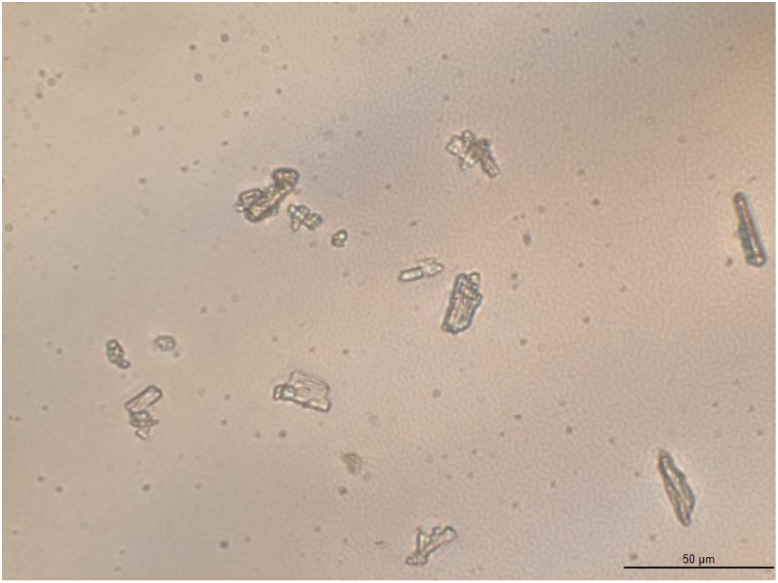
Optical microphotograph of posaconazole API Form I dispersed in mineral oil using transmission mode, brightfield illumination and 40× objective lens (400 magnification).

The morphology of posaconazole Form I was observed via optical microscopy. Columnar and plate translucent particles were detected (Figure 8).

**Figure 9:**
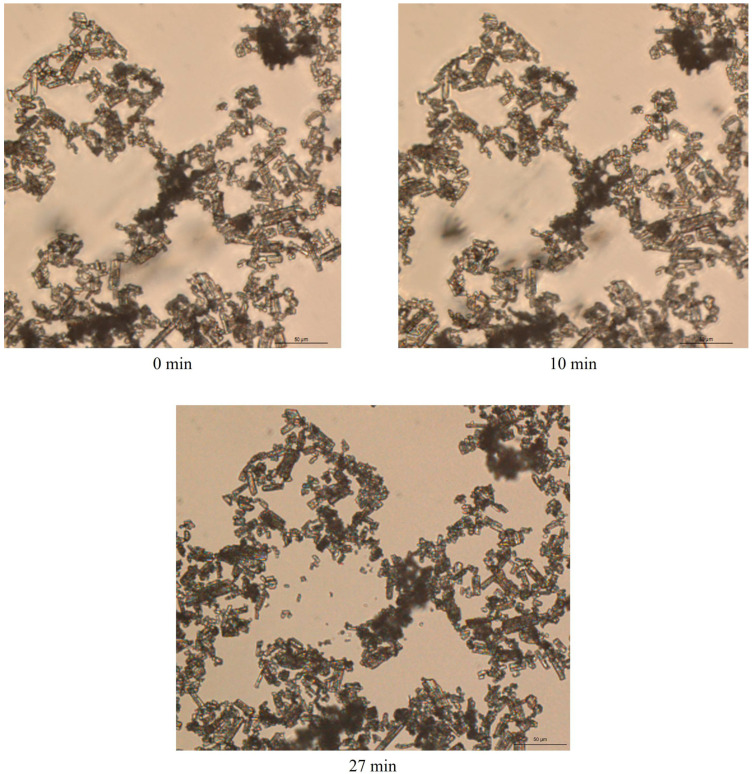
Optical microphotographs of the same field of the grains of posaconazole API Form I 0 min, 10 min and 27 min after being wetted with type II water on an object glass slide using transmission mode, brightfield illumination and 20× objective lens (200 magnification).

In order to further elucidate the behaviour of the posaconazole crystals in the presence of water, some grains of posaconazole Form I were observed after mixing with some drops of type II water (Figure 9). Crystals were found to aggregate but no apparent differences between the crystal shapes of Form I and S were observed. These aggregated particles correspond to posaconazole Form-S. Immediately after wetting Form I (0 min), a net of aggregated columnar and plate crystals was formed (Figure 9). The crystals adhered to each other, while water molecules were trapped among the crystal particles. For the first 25 min, no significant difference in the morphology of the crystals was observed despite the dynamic motions of the particles in the net. However, after 26 min, enhanced dynamic motions were observed, as water molecules were evaporating, leading to some crystal rearrangement in the net. More crystal particles were concentrated in the net during water evaporation. As water was evaporating, the particles at the edges of the sample came closer to each other and to the centre of the sample entrapping more water molecules in the internal of the net, as the particles were trying to retain the crystal net. The intensity of this phenomenon increased as more water molecules evaporated from the sample. When water was completely evaporated after 27 min, the crystal net was retained although not as initially ordered (Figure 9). The evaporation of water molecules between 26min and 27 min is quick in the external part of the crystal net, while it is delayed in the water molecules trapped in the crystal net. The phenomenon is better observed in the Supplementary Video (Video S1), in which the dynamic motions of the crystal particles during the last minute of evaporation could be clearly detected. This behaviour suggests that in Form-S channels or pores of water are formed among posaconazole crystals.

#### 2.3.5. Thermogravimetric Analysis (TGA) and Differential Scanning Calorimetry (DSC)

Form-S is easily converted to Form I upon dehydration so the DSC thermorgraphs of Form I and Form-S were found to be identical to each other (not shown), as it was expected, with the addition of a water loss broad endotherm at approximately 100 °C in the thermograph of Form-S.

**Figure 10:**
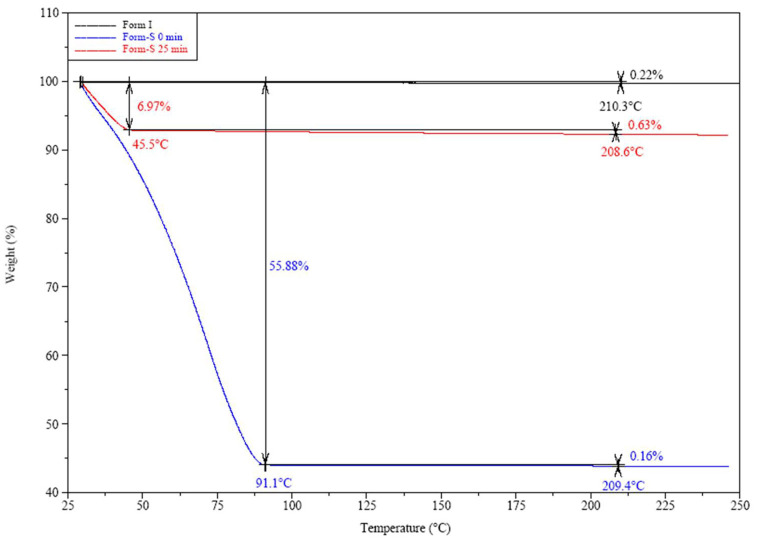
TGA thermograms of posaconazole Form I and posaconazole Form-S immediately after its isolation (0 min) and 25 min after its isolation.

TGA was employed in order to investigate the number of water molecules per posaconazole Form-S molecule. The TGA thermogram of posaconazole Form I revealed that it was, indeed, an anhydrous polymorph of posaconazole, as a weight loss of only 0.22% was determined till 210.3 °C (Figure 10), which corresponded to a loss of 0.09 mol of water. Regarding Form-S, in the TGA thermogram acquired immediately after the end of filtration a water loss of approximately 56% was calculated until a temperature of 91.1 °C, which was attributed to approximately 50 mol water per API mol. This amount of water molecules in the structure was due to the remaining water after filtration of the water dispersion. For this reason, the isolated Form-S was left in ambient temperature to dry for the maximum time that Form-S was stable, i.e., 25 min, before the TGA analysis. In this case, a water loss of 6.97% was determined till a temperature of 45.5 °C and an extra 0.63% was detected from 45.5 °C to 208.6 °C (Figure 10). Thus, a total 7.60% weight loss was determined for Form-S 25 min after the end of filtration. This weight loss corresponded to 3 mol water per mol of posaconazole.

## 3. Discussion

The preparation and isolation of pure posaconazole Form-S was proven to be a challenging process. This was because Form-S is prone to conversion to the thermodynamically stable Form I in ambient conditions, hence the detection of pure Form-S was limited due to available handling time. In addition, the wettability of posaconazole Form I was found to be low and thus the inadequate wetting of the API particles led to the partial conversion of posaconazole Form I to Form-S.

The only method which was able to achieve complete conversion to Form-S was that of homogenising a posaconazole aqueous dispersion in a sonication bath for at least 10 min. This method achieved the deaggregation of posaconazole aggregates and thus complete wetting of all particles was possible. All the other tested methods (manual shaking, vortex, and magnetic stirring) led to partial transformation of Form I to Form-S.

This is the first time that the isolation of pure posaconazole Form-S from Form I is described. In the literature only the XRPD pattern of posaconazole Form-S could be found in a patent, as produced from benzylated posaconazole [26]. However, Form-S was not further characterised in any other study. In our study, the Raman spectrum, the ATR spectrum and the thermogram from TGA analysis are presented for the first time. From these results, it was found that Form-S is a hydrate form of posaconazole. In addition, through optical microscopy, it was found that posaconazole Form-S is a hydrate form, where particles form a net of loosely connected crystals, with water channels or pores among the particles. The net is stabilised through intermolecular forces among posaconazole molecules and between posaconazole molecules and water molecules.

In this study, the prominent role of XRPD in the discrimination of the two polymorphs as APIs was verified, although Raman spectroscopy could be used as an alternative for the differentiation between posaconazole Form I and posaconazole Form-S APIs. ATR spectroscopy could also be used for the identification of posaconazole polymorphism, but the wide peaks corresponding to water in the spectrum may overlap crucial peaks for the differentiation of the two polymorphs. TGA analysis revealed that posaconazole Form-S is a hydrate form with three water molecules. Therefore, the combination of XRPD, a spectroscopic technique between Raman and ATR spectroscopy and a thermal analytical technique, such as TGA, could lead to the identification of posaconazole as the pseudopolymorphic hydrate Form-S.

## 4. Materials and Methods

### 4.1. Materials and Samples

Posaconazole API Form I (MSN Laboratories, Kondapur, India) and all posaconazole oral suspensions excipients, i.e., polysorbate 80, sodium citrate monohydrate, citric acid monohydrate, simethicone, xanthan gum, sodium benzoate, liquid glucose, glycerin, artificial cherry flavour and titanium dioxide, were kindly provided by the Greek pharmaceutical company GENEPHARM S.A (Pallini, Greece).

### 4.2. The Isolation of Pure Form-S

Different methods for the production of pure posaconazole Form-S were tested. In all cases, aqueous dispersions of posaconazole were prepared by mixing posaconazole API Form I and purified type II water (15 MΩ·cm at 25 °C, Elix, Merck Millipore, Darmstadt, Germany) in a final concentration of 40 mg/mL. The methods were differentiated in the way of mixing the API with water. Manual shaking, vortexing at 2500 min^−1^ (MS2 Minishaker IKA^®^, IKA^®^-Werke GmbH & Co. KG, Staufen, Germany), magnetic stirring at 250 rpm, 500 rpm, 750 rpm and 1000 rpm (Hanna^®^ HI 190M, HANNA instruments, Woonsocket, RI, USA) and sonication at 42 kHZ ± 6% (100 W) (Branson 2510E-MT, Branson Ultrasonics Corporation, Danbury, CT, USA) were employed for this purpose. Among all these methods, sonication for 15 min was selected to ensure homogenisation of the aqueous dispersions.

For the isolation of Form-S from the aqueous dispersions, centrifugation of 5 mL posaconazole dispersion at 8000 rpm and 25 °C for 23 min (Heraeus Biofuge Stratos, Kendro, Osterode, Germany) and filtration under vacuum of 5 mL posaconazole dispersion using circular nitrocellulose filters with 25 mm diameter (MF-Millipore^®^ Membrane Filter, 0.22 µm pore size, filter code: GSWP02500, Merck KGaA, Darmstadt, Germany) and a vacuum pump (KNF Neuberger Inc. Laboport, Trenton, NJ, USA) were tested. Of the two separation methods, filtration was selected because of its rapidity and simplicity. In order to delay the polymorphic transition to Form I, the isolated precipitate was covered with a 6 μm LDPE cling film (Vileda Freshmate^®^ 50 m, FHP Hellas, Kifisia, Athens, Greece).

### 4.3. X-ray Powder Diffraction (XRPD)

For the identification of posaconazole polymorphism, an X-ray powder diffractometer (Bruker D2 Phaser 2nd Gen, Bruker, Karlsruhe, Germany) was used, equipped with a standard Bragg Brentano geometry with a fixed primary and linear LYNXEYE (1D mode) detector. As the incident radiation in the anode, a ceramic X-ray tube KFL Cu-2K, 0.4 mm × 12 mm with Ka spectral line (λ = 1.54184 Å) was used. The scan mode was continuous and a locked coupled scan type was used. The source voltage was set at 30 kV and the current at 10 mA; thus, the tube was working with 300 W. The divergence slit was regulated at 0.6 mm, the receiving slit at 8 mm, the soller slit at 2.5° and the air scatter screen at 3 mm. The Position Sensitive Detector (PSD) opening value was 5° 2-theta. The step size was set at 0.02° 2-theta and the region of 4°–40° 2-theta was scanned. No rotation was applied in any of the samples.

Variable scan rates were used. XRPD patterns were recorded of all excipients, pure posaconazole Form I, the isolated Form-S and the mixtures of Form I with the excipients, at a scan speed of 1.0 s/step. For recording the XRPD patterns of pure posaconazole Form I and pure posaconazole Form-S for the characterisation of the novel polymorph, a slow scan speed of 4.0 s/step was employed. However, when the stability of pure Form-S was studied, a very fast scan rate of 0.2 s/step was used. The samples were spread on polymethylmethacrylate (PMMA) XRPD sample holders with a 25 mm diameter and 0.5 mm depth circular cavity. The background of the XRPD patterns was subtracted using the software DIFFRAC.EVA.V6.0 (Bruker, Karlsruhe, Germany).

### 4.4. Micro-Raman Spectroscopy

A portable Raman spectrometer (iRaman Plus BWS465-785H, B&W Tek Inc., Newark, DE, USA) equipped with an optic microscope (B&W Tek Inc., Newark, DE, USA) and a laser with a 785 nm excitation line was used. An Olympus objective lens (20×) was employed for focusing onto the sample surface. The system was equipped with a high quantum efficiency CCD array detector with deeper cooling and a high dynamic range. The nominated power of the incident laser was 455 mW and 10% of the laser power was used for recording the Raman spectra of posaconazole polymorphs. The time of each scan was set at 20 s and each spectrum was the result of 3 accumulated scans. Each spectrum was recorded in the region of 65–2800 cm^−1^ with a resolution <3.5 cm^−1^ at 912 nm. The Raman spectra were recorded using the software BwSpec4^®^ (B&W Tek Ink, Newark, DE, USA).

Posaconazole Form I was spread on a glass slide coated with gold substrate (EMF Corporation, Ithaca, NY, USA) with a spatula. The gold slide dimensions were 2.5 cm × 7.5 cm with 1.0 mm thickness. A binding layer of titanium (50 Å) was deposited under the gold layer (1000 Å), which serves as a reflectance surface [44]. For the acquisition of the micro-Raman spectrum of posaconazole Form-S isolated after filtration, the precipitate was placed on the 1-well cavity of a glass slide coated with gold substrate and it was covered with the 6 μm LDPE cling film for the delay of the polymorphic transition. The cavity’s diameter was 1.5 cm and its depth 0.6 mm, while the dimensions of the slide were 2.6 cm × 7.6 cm with 1.25 mm thickness.

### 4.5. Attenuated Total Reflection (ATR) Spectroscopy

A Fourier-Transform Infrared (FTIR) spectrometer (PerkinElmer Spectrum 100, PerkinElmer Inc., Waltham, MA, USA) equipped with an Universal ATR Sampling Accessory with a 9-bounce crystal diamond ZnSe was used to obtain the ATR spectra of posaconazole polymorphs. The ATR spectra were acquired in the region of 4000–650 cm^−1^ with a spectral resolution of 4 cm^−1^. Each spectrum was a result of 10 accumulated scans. The ATR spectra were acquired using the software Spectrum^®^ 6.3.5 (PerkinElmer Inc., Waltham, MA, USA). For posaconazole Form I, an adequate quantity was placed with a spatula on the ATR crystal and a cotton swab was used for pressing the sample. Similarly, a quantity of approximately 20 mg of the isolated posaconazole precipitate was spread on the ATR crystal with a spatula and it was covered with the LDPE cling film.

### 4.6. Optical Microscopy

An optical microscope (Leica DM 2500M, Leica Microsystems Ltd., Heerbrugg, Switzerland) was used to observe posaconazole API Form I and posaconazole Form-S. The optical microscope was equipped with a digital camera (Leica DFC420 C, Leica Microsystems Ltd., Heerbrugg, Switzerland) with Peltier cooling system and a 5-megapixel CCD Bayer Array RGB filter capturing high-resolution images. The optical microscope was equipped with a condenser with numerical aperture (NA) of 0.90, which was part of a phase contrast turret condenser of 6-positions and eyepieces offering 10× magnification (HC PLAN S, Leica Microsystems Ltd., Heerbrugg, Switzerland). For observing posaconazole polymorphs, the 40× objective lens (NA 0.75) (HCX PL FLUOTAR PH2, Leica Microsystems Ltd., Heerbrugg, Switzerland) was selected. Thus, a total magnification of 400 was achieved. Transmitted light microscopy was applied on the samples using brightfield illumination. The software LAS^©^ V4.13 (Leica Microsystems Ltd., Heerbrugg, Switzerland) was used for capturing the images of the polymorphs.

For observing posaconazole Form I, 5 mg API were dispersed in 5 mL mineral oil (ACRŌS ORGANICS, Geel, Belgium) and a drop of the sample was placed on a 76 × 26 × 1 mm microscope slide (Paul Marienfeld GmbH & Co. KG, Lauda-Königshofen, Germany) with a 22 × 22 mm cover slip (Paul Marienfeld GmbH & Co. KG, Lauda-Königshofen, Germany) covering the sample.

For observing posaconazole Form-S, a 40 mg/mL aqueous dispersion of posaconazole Form I was prepared by homogenising the sample for 15 min using a sonication bath. The dispersion was diluted to a final concentration of approximately 1 mg/mL. A drop of this sample was placed on a microscope slide which was covered with a cover slip.

For monitoring the conversion of posaconazole Form-S to Form I, a few grains of posaconazole Form I were placed on the microscope slide and a drop of purified water was added to disperse the API with the assistance of a spatula. No cover glass was placed on the sample in order to observe posaconazole API’s behaviour while water molecules were evaporating. The multi-time lapse and video tools were used to capture the image of the same field for 27 min with 1 min time intervals.

### 4.7. Thermogravimetric Analysis (TGA)

A TGA Q50 (TGA Q50 V5.3 Build 171, TA Instruments, Newcastle, DE, USA) was used for obtaining the thermograms of posaconazole Form I and posaconazole Form-S. The instrument possesses an isothermal temperature accuracy of ±1.0 °C, an isothermal temperature precision of ±0.1 °C, a balance sensitivity of 0.1 μg and a weighing precision of ±0.01%. Approximately 10 mg of each polymorph was loaded in platinum sample pans. The thermograms were acquired at a temperature range of 25–250 °C with a heating rate of 10 °C/min in a nitrogen (N_2_) atmosphere, with a flow rate of 40.0 mL/min. The software THERMAL ADVANTAGE 3.2.0Q100 (TA Instruments, Newcastle, DE, USA) was used for the acquisition of the TGA thermograms.

### 4.8. Conatct Angle

The sessile drop method was applied to three different discs of posaconazole Form I, which were pressed at 2 tons for 2 min (Specac Atlas^®^ Manual Hydraulic Press 15T, Specac Ltd., Orpington, England, UK). The discs were stored at 100% RH for 24 h and then they were placed on a glass frit in touch with water, so that all pores of the discs would be saturated with humidity [36]. The contact angle of purified water on posaconazole Form I discs was determined through Image J software (National Institutes of Health, Bethesda, MD, USA and the Laboratory for Optical and Computational Instrumentation (LOCI, University of Wisconsin), Madison, WI, USA) in triplicates after placing 5 μL ultra-pure water on each disc and capturing an image 10 s later.

## 5. Conclusions

The employment of XRPD, Raman spectroscopy, ATR, TGA and optical microscopy lead to elucidation of the posacozanole Form-S structure. It was found that Form-S is a hydrate form, with approximately three molecules of water per API molecule. The unit cells of both Form-S and Form I were found to be in the monoclinic system, with Form-S being a deformed monoclinic lattice with a somewhat orthorhombic shape. From optical microscopy it was also found that Form-S crystals of posaconazole regroup in order to form pores or channels around water molecules. Form-S is an unstable form that is transformed to Form I upon dehydration. This process takes approximately 30 min in ambient conditions.

## Figures and Tables

**Table 1:** The constituents of posaconazole oral suspensions and their concentration.

Constituents	Role	Concentration (mg/mL)
Posaconazole Form I	API	40.0
Polysorbate 80	Non-ionic surfactant	15.0
Simethicone	Anti-foaming agent	3.0
Sodium Benzoate	Preservative	2.0
Xanthan Gum	Suspending agent	3.0
Glycerol	Co-solvent Sweetening agent	100.0
Liquid Glucose	Sweetening agent	300.0
Titanium Dioxide	Opacifier agent	4.0
Artificial Cherry Flavour	Flavouring agent	5.0
Citric Acid Monohydrate	Buffering agent	1.5
Sodium Citrate Dihydrate	Buffering agent	0.6
Purified Water	Solvent	q.s. 1.0 mL

**Table 2:** The results of the polymorphic conversion of posaconazole Form I to Form-S in 40 mg/mL aqueous dispersions prepared by different sample preparation methods.

Sample Preparation Method	Posaconazole Polymorphism
Manual Shaking 1 min	Form-S with traces of Form I
Manual Shaking 5 min	Form-S with traces of Form I
Vortex 1 min	Form-S with traces of Form I
Vortex 2 min	Form-S (lack of repeatability)
Vortex 5 min	Form-S with traces of Form I
Magnetic Stir 250 rpm 30 min	Form-S with traces of Form I
Magnetic Stir 500 rpm 30 min	Form-S with traces of Form I
Magnetic Stir 750 rpm 30 min	Form-S with traces of Form I
Magnetic Stir 1000 rpm 30 min	Form-S (lack of repeatability)
Magnetic Stir 1000 rpm 5 min	Form-S and Form I
Magnetic Stir 1000 rpm 15 min	Form-S with traces of Form I
Magnetic Stir 1000 rpm 60 min	Form-S with traces of Form I
Sonication 1 min	Form-S and Form I
Sonication 2 min	Form-S with traces of Form I
Sonication 5 min	Form-S and Form I
Sonication 10 min	Form-S
Sonication 15 min	Form-S

**Table 3:** Comparison of the crystallographic data of posaconazole Form I and Form-S, as determined via the dichotomy method using PreDICT software, applied on their XRPD patterns. The angles α and γ are 90.0°.

Crystallographic Data	Form I	Form-S
Crystal System	Monoclinic	Monoclinic
Bravais Crystal Lattice	Simple Monoclinic	Simple Monoclinic
M_20_ (de Wolff)	207.4	24.3
F_20_ (Smith-Snyder)	505.5 (0.0009, 43)	74.00 (0.0079, 34)
Edge a	(12.536 ± 0.001) Å	(12.380 ± 0.005) Å
Edge b	(6.348 ± 0.0001) Å	(6.305 ± 0.003) Å
Edge c	(22.780 ± 0.001) Å	(23.126 ± 0.016) Å
Angle β	96.387° ± 0.002°	93.140° ± 0.034°
Unit Cell Volume (V)	(1801.48 ± 0.10) Å^3^	(1802.47 ± 1.68) Å^3^

**Table 4:** Differences between the Raman spectra of posaconazole Form I and posaconazole Form-S.

Raman Shift (cm^−1^)	Posaconazole Form I	Posaconazole Form-S
701–714 cm^−1^	Medium double peak at 701 cm^−1^ and 714 cm^−1^	Shoulder at 702 cm^−1^ and medium peak at 712 cm^−1^
729–745 cm^−1^	Shoulder at 729 cm^−1^ and strong peak at 745 cm^−1^	Single strong peak at 738 cm^−1^
785–798 cm^−1^	Strong double peak at 785 cm^−1^ and 796 cm^−1^	Shoulder at 789 cm^−1^ and strong peak at 798 cm^−1^
820–835 cm^−1^	Broad shoulder at 823 cm^−1^ followed by a medium peak at 835 cm^−1^	Medium double peak at 820 cm^−1^ and 835 cm^−1^
850 cm^−1^	Weak single peak at 850 cm^−1^	End of a shoulder due to the peak at 835 cm^−1^
911 cm^−1^	Shoulder at 911 cm^−1^ at the descent of the single peak at 900 cm^−1^	No shoulder and no peak at 911 cm^−1^
941–966 cm^−1^	Weak peak at 941 cm^−1^ followed by a strong peak at 966 cm^−1^	Broad shoulder at 941 cm^−1^ at the ascent of the strong peak at 966 cm^−1^
1135–1147 cm^−1^	Medium single peak at 1135 cm^−1^ followed by a shoulder at 1147 cm^−1^	Medium single peak at 1139 cm^−1^ without shoulder at 1147 cm^−1^
1205–1224 cm^−1^	Strong double peak at 1205 cm^−1^ and 1222 cm^−1^	Strong double peak at 1208 cm^−1^ and 1224 cm^−1^
1264–1287 cm^−1^	Strong peak at 1278 cm^−1^ with two shoulders at 1268 cm^−1^ and 1287 cm^−1^	Strong peak at 1274 cm^−1^ with only one shoulder at 1264 cm^−1^
1328–1350 cm^−1^	Strong double peak at 1329 cm^−1^ and 1350 cm^−1^	Strong double peak at 1328 cm^−1^ and 1346 cm^−1^
1382–1402 cm^−1^	Strong double peak at 1382 cm^−1^ and 1402 cm^−1^ The peak at 1382 cm^−1^ is of higher intensity	Shoulder at 1389 cm^−1^ and strong single peak at 1402 cm^−1^ The peak at 1402 cm^−1^ is of higher intensity
1444–1460 cm^−1^	Shoulder at 1444 cm^−1^ and medium peak at 1460 cm^−1^	Medium double peak at 1444 cm^−1^ and 1460 cm^−1^

**Table 5:** Differences between the ATR spectra of posaconazole Form I and posaconazole Form-S.

Wavenumber (cm^−1^)	Posaconazole Form I	Posaconazole Form-S
3800–2700 cm^−1^	Multiple weak peaks	Very strong broad peak with centre at 3370 cm^−1^ corresponding to water
1770–1570 cm^−1^	Strong single peak at 1686 cm^−1^ and weak double peak at 1618 cm^−1^ and 1603 cm^−1^	Strong broad peak with centre at 1638 cm^−1^, corresponding to water and overlapping the peaks at 1686 cm^−1^, 1618 cm^−1^ and 1603 cm^−1^
1552–1549 cm^−1^	Medium single peak at 1549 cm^−1^	Medium single peak at 1552 cm^−1^
1483 cm^−1^	Very weak single peak at 1483 cm^−1^	No peak
1427–1419 cm^−1^	Weak peak at 1427 cm^−1^ with shoulder at 1419 cm^−1^	Weak single peak at 1419 cm^−1^
1397–1393 cm^−1^	Medium single peak at 1397 cm^−1^	Medium single peak at 1393 cm^−1^
1307–1290 cm^−1^	Ascent at 1307 cm^−1^ of the weak double peak at 1298 cm^−1^ and 1290 cm^−1^	Weak double peak at 1307 cm^−1^ and 1297 cm^−1^
1277–1270 cm^−1^	Medium peak at 1277 cm^−1^ with shoulder at 1270 cm^−1^	Medium single peak at 1272 cm^−1^
1135 cm^−1^	Strong and sharp single peak at 1135 cm^−1^	Medium broad single peak at 1135 cm^−1^
1107–1099 cm^−1^	Medium peak at 1107 cm^−1^ with shoulder at 1099 cm^−1^	Medium single peak at 1105 cm^−1^
1083 cm^−1^	Weak single peak at 1083 cm^−1^	No peak
1075–1057 cm^−1^	Medium broad double-like peak at 1069–1059 cm^−1^	Medium double peak at 1075 cm^−1^ and 1057 cm^−1^, stronger at 1057 cm^−1^
1041–1035 cm^−1^	Weak double peak at 1041 cm^−1^ and 1035 cm^−1^	Weak single peak at 1040 cm^−1^
1022–1012 cm^−1^	Medium single peak at 1017 cm^−1^	Medium peak at 1012 cm^−1^ with shoulder at 1022 cm^−1^
900–650 cm^−1^	Multiple strong, medium and weak peaks Medium single peak at 870 cm^−1^ Weak single peak at 776 cm^−1^	Ascent of a very broad peak, corresponding to water, with weak overlapped peaks of Form-S No peaks were detected at 870 cm^−1^ and 776 cm^−1^ even after 20 min drying

## Data Availability

Not applicable.

## Supplementary Materials

The following supporting information can be downloaded at: https://www.mdpi.com/article/10.3390/ph16010065/s1, Video S1: Video of the grains of posaconazole API Form I between 26 and 27 min after being wetted with type II water on an object glass slide in the transmission mode, brightfield illumination and 20× objective lens (200 magnification).
